# MedXFit—Effects of 6 months CrossFit® in sedentary and inactive employees: A prospective, controlled, longitudinal, intervention study

**DOI:** 10.1002/hsr2.749

**Published:** 2022-08-07

**Authors:** Tom Brandt, Annette Schmidt, Timo Schinköthe, Elisabeth Heinz, Yannik Klaaßen, Selina Limbara, Marian Mörsdorf

**Affiliations:** ^1^ Institute of Sports Science, Department of Human Sciences University of the Bundeswehr Munich Neubiberg Germany; ^2^ Comprehensive Cancer Center Munich CCCLMU Munich Germany

**Keywords:** exercise, fitness, functional movement, health, high‐intensity interval training, military

## Abstract

**Background and Aims:**

Sedentary behavior and physical inactivity are associated with musculoskeletal disorders (MSD). Muscle and mobility enhancing training is recommended to promote musculoskeletal fitness and prevent MSD. A functional fitness program emphasizing the importance of musculoskeletal fitness is provided by CrossFit®. However, data from long‐term CrossFit® interventions assessing measures of musculoskeletal fitness in sedentary and inactive individuals does not exist.

**Methods:**

Thi**s** prospective, controlled study investigates the effects of 6 months CrossFit® training (2×60 min/week) in inactive adults (in terms of <2 muscle or mobility enhancing training sessions per week) with predominantly sitting or standing occupations. 91 participants were initially assessed, 2 were excluded, 55 self‐selected for intervention (IG), and 34 for the control group (CG). Primary endpoint was a change in mobility (Functional Movement Screen score). Secondary endpoints were changed in strength (maximum isometric strength in kg; Dr. Wolff BackCheck®), and well‐being (WHO‐5 score). Key exploratory endpoints were changes in back‐issue measures (pain intensity, limitation, and frequency).

**Results:**

39 participants of IG and 31 of CG completed the evaluation after 6 months. The IG improved significantly more (*p* < 0.001) compared with the CG in the FMS (*η*² = 0.58), trunk extension (*η*² = 0.46), trunk flexion (*η*² = 0.47), trunk lateral flexion left (*η*² = 0.41), trunk lateral flexion right (*η*² = 0.42), upper body push (*η*² = 0.4), upper body pull (*η*² = 0.25), hip extension left (*η*² = 0.18), and hip extension right (*η*² = 0.4). Change of WHO‐5 scores did not significantly differ between groups (*p* = 0.55; *η*² = 0.01). Exploratory analysis of back‐issue data showed a higher decrease for pain intensity, limitation, and frequency in the IG compared with the CG.

**Conclusion:**

This study proves for the first time within the scope of a prospective, controlled study the broad benefits of CrossFit® in inactive adults doing predominantly sedentary work.

## 1 INTRODUCTION

Inadequate physical activity is considered as an important risk factor for chronic diseases like obesity, type 2 diabetes, cardiovascular diseases, and musculoskeletal disorders (MSD).^1^, ^2^ Modern working conditions play an important role regarding sedentary and inactive behavior. According to a nationwide German study (*N* = 18,026) 47.5% of women and 47.2% of men at the age of 18–64 stated to predominantly sit or stand during their working hours. The proportion increases with higher educational levels.^3^ An active lifestyle and less sedentary behavior during leisure time could minimize negative effects.^4^ However, another study (*N* = 22,959) states that only 42.6% of women and 48% of men at the age of ≥18 meet the minimum level of ≥ 150 min of moderate‐intensity aerobic physical activity recommended by the WHO.^5^ Muscle enhancing activity recommendations (≥2 workouts/week) are met by 27.6% of women and 31.2% of men.^6^

As mentioned above this may support the development of several diseases. Especially MSD has to be mentioned here. In a study concerning the health status of adults 57.9% of women and 52.2% of men reported joint pain in the past 12 months.^7^ Furthermore, MSD alone is responsible for about 25% of sick days,^2^ are associated with a lower quality of life, and may accelerate the loss of functional capacity below the disability threshold.^8^ Aside from that, an intact musculoskeletal system (strength, coordination, and flexibility) is the foundation to stay physically mobile and train other physiologically important systems (e.g., cardiopulmonary and nervous system). While strength,^9^, ^10^ coordination,^11^ and flexibility^12^ deteriorate with age, physical training has widely been proven to slow down this decline and keep the musculoskeletal system intact.^13^

Based on those findings functional strength and conditioning programs embedded in corporate health management programs might help to keep the musculoskeletal system intact, hinder the development of noncommunicable diseases and reduce sick days in physically inactive employees doing predominantly sedentary work. A time‐efficient training system that covers the recommendations of the WHO and American College of Sports and Medicine to improve health is provided by CrossFit® (CF).^4^, ^5^ CF is a high‐intensity, functional fitness program. What stands out about CF is the integration of exercises from various disciplines such as weightlifting or gymnastics, which place high demands on strength, coordination, and mobility.^14^

Scientific literature in this field covers the effects of CF on body composition, life and health aspects, psycho‐physiological parameters, psycho‐social behavior, and the risk for musculoskeletal injuries.^15^ Positive effects of CF have been found in six fitness domains (cardiovascular/respiratory endurance, stamina, strength, flexibility, power, and balance).^16^, ^17^, ^18^ Injuries in CF (3.1 injuries/1000 h of training) occur as often as in weightlifting, powerlifting, gymnastics, and fitness training but less often than in contact sports like rugby.^19^ According to an online survey 19.4% of 386 CF athletes got injured in a 6‐month period, with shoulder (25%) and low back injuries (14.3%) being the most common.^20^ Nevertheless, only a few studies on CF with a high level of evidence and low risk of bias currently exist.^15^ Data regarding long term effects of CF on the musculoskeletal system in populations that have an increased risk for developing noncommunicable diseases (e.g., MSD) have yet to be collected.

Therefore, the aim of this study was to investigate whether 6 months of CF training improve mobility (primary endpoint), strength (secondary endpoint), and well‐being (secondary endpoint) in inactive individuals (in terms of <2 muscle and/or mobility enhancing training sessions per week prior study participation) with a predominantly sitting or standing occupation. Furthermore, back issues were assessed for exploratory purposes.

## 2 MATERIALS AND METHODS

### 2.1 Trial oversight

This study followed a prospective, longitudinal intervention design with control (CG) and intervention group (IG). Data were collected from October 2020 to August 2021. Both groups were tested in the same manner at baseline (t0) and after 6 months (t1) and self‐selected for either IG or CG. Participants of the CG were instructed to maintain their current activity level. This was checked via questionnaire at t1. Nonfulfillment of inclusion criteria (≥ 2 mobility or muscle enhancing training sessions per week; work not mostly sitting or standing anymore) led to exclusion from the study. The IG attended a CF program at the military affiliation CF Kokoro®. The study was integrated in the corporate health management of the University of the Bundeswehr Munich (UniBw M). Participants were allowed to train during their working hours.

**Figure 1:**
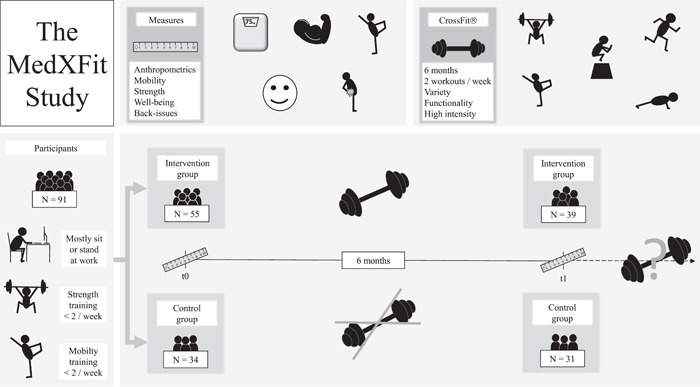
Schematic overview of the MedXFit study.

The Institutional Ethics Committee of the UniBw M approved the study protocol, ensuring that it conformed to the ethical guidelines of the 1975 Declaration of Helsinki. Informed consent was obtained from all subjects involved in the study. The trial was registered on ClinicalTrials.gov with the trial number NCT05109286. An overview of the study design is displayed in Figure 1.

### 2.2 Participants

Military and civilian personnel of the UniBw M (age = 18–65 years, male and female) was invited to participate in the study by flyer and email. Inclusion criteria were a predominantly sitting or standing occupation and inactivity in terms of participation in less than two muscle and/or two mobility enhancing training sessions per week. Exclusion criteria were pregnancy and health issues that would disqualify for participation in regular exercise and the applied tests (severe injuries to the musculoskeletal system, osteoporosis, intervertebral disc damage, joint replacements, hypertension, and fresh scars). Inclusion and exclusion criteria were checked via questionnaires at t0 and t1.

Demographics and anthropometrics of initially assessed participants are displayed in Table 1.

**Table 1:** Demographics and anthropometrics of initially assessed participants (t0)

Variable	Control group	Intervention group	*p*
*N*	34	57	
Gender			0.83
Male (*N*)	41.2% (14)	45.6% (26)	
Female (*N*)	58.8% (20)	54.4% (31)	
Diverse	0%	0%	
Age (years), mean (SD)	36.7 (11.4)	38.2 (12.1)	0.55
BMI (kg/m^2^), mean (SD)	27.3 (5.3)	25.4 (3.9)	0.07
Smoker			0.86
Yes (N)	11.8% (4)	10.5% (6)	
No (N)	88.2% (30)	89.5% (51)	

Abbreviations: BMI, body mass index; SD, standard deviation.

When participants were not able to attend the test session t1 in the laboratory (e.g., sickness, remote work, and quarantine) surveys were done by telephone.

### 2.3 Training intervention

CF is a strength and conditioning program that attempts to improve physical competence in 10 fitness domains (cardiovascular/respiratory endurance, stamina, strength, flexibility, power, speed, coordination, agility, balance, and accuracy). Therefore, CF prescribes constantly varied, high‐intensity functional movements. Despite similarities to actual sports (monitoring time, distance, repetitions, and maximum lifted weights) CF stresses the importance of proficient technical exercise execution to achieve high levels in safety, efficacy and efficiency.^14^

The IG committed to attend two training sessions per week for 6 months. These were 60‐min‐group‐sessions supervised by a coach. Participants had to sign in for each session via online schedule. Registrations were tracked.

The training format included 0–10 min introduction and warm‐up, 5–10 min mobility training, 5–30 min skill and/or strength training, 5–30 min of high‐intensity training, and 5–10 min cool down. A sample training session for every week is provided in Table S1. During Weeks 1–10, the main goal was to develop technical proficiency in fundamental movement patterns like getting up off the ground, squatting, lifting objects off the ground, upper body push and pull movements, and carrying objects for distance while maintaining a stable core. Thus, a high proportion of the sessions was mobility and skill‐based. The strength and high‐intensity aspect of the sessions increased steadily after the first 10 weeks. Nevertheless, mobility and skill development were continually done in each session. While all participants followed the same program structure, training parameters were adjusted throughout the program according to each person´s fitness level.

A maximum of 10 participants per session was set during the first 2 months. In the third month, an online course was implemented and held live 2–5 times a week while in COVID‐19 lockdown. During online sessions, typical gym equipment (e.g., barbells or kettlebells) was replaced by every day (odd) objects and more unilateral movements were integrated. Sessions were recorded and uploaded for those who could not attend the live sessions. Groups of six participants per session were set after the lockdown to meet COVID‐19‐related rules. Online sessions were held parallel for 3 more months to ensure adherence to the training program for participants in the home office. All training sessions were carried out by CF level 1 and level 2 certified trainers of CF Kokoro®.

### 2.4 Endpoints and protocol

Primary endpoint of this study was the change in mobility (Functional Movement Screen score)^21^, ^22^ from t0 to t1. Secondary endpoints were the changes in strength (maximum isometric strength in kg; Dr. WOLFF BackCheck® 617)^23^, ^24^ and well‐being (WHO‐5 score)^25^ from t0 to t1. Back issues (pain intensity, limitation, and frequency) were assessed via a questionnaire (pain intensity and limitation on an 11‐point scale; frequency in days/week) for exploratory purposes.

Both sessions followed an identical protocol. Neither testing personnel nor participants were blinded. All tests were conducted by the same person throughout the study. Test sessions started with a questionnaire to assess the participant's medical history, physical activity, well‐being, and back issues. Afterward anthropometrics, mobility, and strength were measured. Familiarization sessions were not done prior t0. All tests were executed in sportswear without shoes. Both groups were asked to avoid any intensive physical training 24 h prior the test sessions. A breathing mask had to be worn during test sessions due to COVID‐19 pandemic‐related restrictions

#### 2.4.1 Body composition

Height and bodyweight were measured in sportswear without shoes. Height was measured with a SECA® 213 and bodyweight with a TANITA® BC‐545 scale. Measurements were required to calculate strength set points for the Dr. WOLFF BackCheck® 617.

#### 2.4.2 Mobility

The Functional Movement Screen (FMS)^21^, ^22^ was done to evaluate mobility. It consists of seven fundamental movements. These movements are deep squat, hurdle step, inline‐lunge, shoulder mobility, active straight leg raise, trunk stability push up, and rotary stability quadruped. Each movement is rated with a score from 0 to 3 resulting in a maximum total score of 21. Specific movement criteria must be accomplished to score 1–3. If participants report any pain the movement is scored with 0. Additionally, three movements provide a clearing test. Clearing tests are done after the actual movement. If pain is reported during the clearing test the participant scores 0 in the movement no matter what was achieved before. For bilateral movements the lower rated side is counted.^21^, ^22^

#### 2.4.3 Strength

Strength was assessed with the Dr. WOLFF BackCheck® 617 (BC). It allows to measure maximum isometric strength (values are given in kilograms). The BC is high enough in test‐/retest reliability and criteria validity to be used in scientific research.^24^ Participants were instructed and then given three attempts per movement. The best result was selected. Movements were done in the following sequence: trunk extension (TE), trunk flexion (TF), upper body push (UPush), upper body pull (UPull), trunk lateral flexion left (TLFl) and right (TLFr), and hip extension left (HEl) and right (HEr).

#### 2.4.4 Well‐being

Well‐being was measured with the World Health Organization Well‐Being Index (WHO‐5). It consists of five questions that focus on subjective well‐being of participants. Scores range from 0 to 25 (5‐point scale per question; 1 = worst, 5 = best). It has adequate validity and is widely used across different fields. Simplicity and time efficiency are additional advantages of this tool.^25^

#### 2.4.5 Back issues

Back issues were assessed for specific areas (neck, shoulders, upper back, the lower back). At first, participants had to report if they had any issues in the past 6 months in the above‐mentioned areas. Thereafter, they were asked to rate their average pain intensity and limitations on an 11‐point scale (0 = no pain or limitation, 10 = highest imaginable pain or limitation). Furthermore, pain frequency was assessed in days per week suffering from issues in the particular area. For pain intensity, limitation, and frequency the area with the highest value was selected for the analysis.

### 2.5 Statistical approach

This study included exclusively individuals that did less than two muscle and/or mobility enhancing training sessions per week prior the study. Consequently, low baseline FMS scores were expected. According to existing literature reporting high FMS scores among CF participants^26^, ^27^ compared with inactive individuals,^28^, ^29^ a large effect for the primary endpoint was expected. Therefore, 29 participants per group were determined to achieve a power of at least 85% on a two‐sided, 5% significance level. Higher time expenditure for the IG and uncertainty regarding impact of COVID‐19‐related restrictions on training attendance (e.g., quarantine, remote work, and availability of training facilities) led to different determined group sizes. Expected dropout rates were 15% (*N*_dropout_/*N*_baseline)_ in the CG and 45% (*N*_dropout_/*N*_baseline)_ in the IG, resulting in 34 and 55 recruited participants.

As the effectiveness of the intervention regarding the primary and secondary endpoints should be determined by the difference in change between groups, a mixed model analysis of variance (ANOVA) was conducted. Change within groups was calculated by subtracting t0 from t1 values. Normal distribution was checked with Q‐Q‐plots and Kolmogorov–Smirnov test. As assumptions for the mixed model ANOVA were not met for every variable (Table S2), two‐sided bootstrapped (bias‐corrected; samples *N* = 1000) independent *t*‐test and Mann–Whitney *U* test on differences in change (t1 − t0) between groups was conducted to support ANOVA results (Tables S3 and S4). Bias‐corrected and accelerated bootstrap method (bootstrap samples *N* = 1000) was applied to give more reliable estimates for the 95% confidence intervals (CI). Statistical significance was set at *p* ≤ 0.05.

The occurrence of back issues was assessed for exploratory purposes. Change values of both groups were calculated by subtracting t0 from t1 values. Mann–Whitney *U* test was conducted to analyze the difference in change between groups.

Values for t0 and t1 as well as changes from t0 to t1 within groups are expressed as mean (standard deviation [SD]). Differences in change between groups are presented as mean (95% CI). Effect sizes of primary and secondary endpoints are given in partial *η* ^2^. Pearson's r was calculated for exploratory endpoints. Data analysis was done with SPSS 28® (IBM SPSS).

## 3 RESULTS

**Figure 2:**
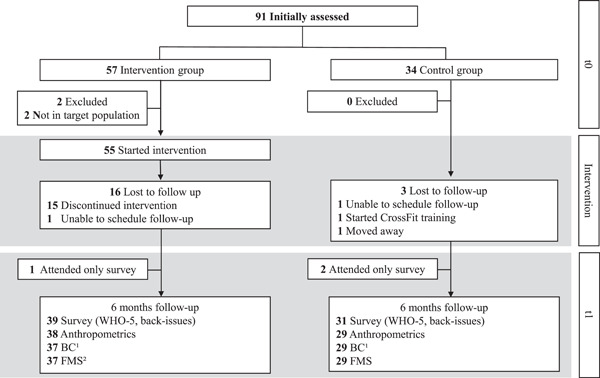
Participant flow over the course of the study.^1^ Due to minor shoulder injuries, two participants of the intervention and one of the control group had to leave out particular movements of the BackCheck®. Additionally, one participant was incapable of completing the BackCheck® as prescribed.^2^ Due to a minor shoulder injury one participant did not complete the full Functional Movement Screen.

Of 91 participants initially screened, two did not meet the inclusion criteria as they already did more than two muscle‐enhancing training sessions per week prior to intervention. After 6 months 39 and 31 data sets were collected from IG and CG. Dropout in the IG (29%; *N*_drop‐out_/*N*_baseline_). was higher than in the CG (9%; *N*_drop‐out_/*N*_baseline_). Data sets were incomplete as one participant of the IG and two of the CG did not attend the follow‐up in person but per telephone. In addition, two participants of the IG and one of the CG suffered from minor shoulder injuries (not related to intervention) at t1. Consequently, not all BC and FMS measures were taken from these individuals. A mean training attendance of 38.1 (8.3) sessions over the course of 26 weeks were documented for participants that completed the intervention phase. Further information regarding the number of participants is shown in Figure 2.

### 3.1 Primary endpoint

Mean FMS scores differed between CG (11.1 [2.8]) and IG (10.4 [2.6]) at t0. The difference did not reach statistical significance (*p* = 0.35). Mean change in FMS score from t0 to t1 was −0.6 (2.4) for CG and 4.6 (2.1) for IG. The difference in change between groups was significant (5.2 [4.1–6.3], *p* < 0.001) and resulted in the highest effect size among all primary and secondary endpoints (*η*² = 0.58).

### 3.2 Secondary endpoints

**Figure 3:**
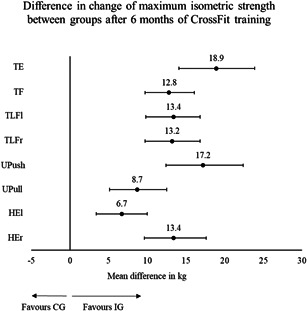
Strength: difference in change between groups from baseline (t0) to 6 months (t1). HEl, hip extension left; HEr, hip extension right; TE, trunk extension; TF, trunk flexion; TLFl, trunk lateral flexion left; TLFr, trunk lateral flexion right; UPush, upper body push; UPull, upper body pull

The IG had higher maximum isometric strength values for all tested movements at t0. For TLFr the difference was significant (*p* = 0.047). After 6 months, the IG improved significantly (*p* < 0.001) more than the CG in all tested movements. The largest effect was observed for TF (*η*² = 0.47) followed by TE (*η*² = 0.46). For TLF similar differences in change were observed for the left (*η*² = 0.41) and right (*η*² = 0.42) side. Upper body pushing strength was higher than pulling strength at t0 in both groups. While the IG improved, the CG worsened in the UPush, resulting in a significant difference in change between groups (*η*² = 0.4). The difference in change in the UPull was lower but significant nonetheless (*η*² = 0.25). The smallest difference in change among all tested movements was observed for HEl (*η*² = 0.18). However, the difference for HEr was higher (*η*² = 0.4). Differences in change values between IG and CG are displayed in Figure 3.

Mean WHO‐5 scores of the CG were higher at t0 compared to the IG but did not reach statistical significance (*p* = 0.26). The mean change in WHO‐5 score from t0 to t1 was higher in the IG (1.8 [3.7]) compared with the CG (1.3 [3.6]). However, no significant difference in change between groups was observed (*p* = 0.55, *η*² = 0.01). The main effect for time was significant (*p* < 0.001, *η*² = 0.15). Values for mobility, strength, and well‐being at t0 and t1 as well as changes within and between groups are presented in Table 2.

**Table 2:** Primary and secondary endpoints after 6 months for intervention and control group

	t0 (baseline)	t1 (after 6 months)	Change within groups	Difference of change between groups	*p*	*η*²
Primary endpoints						
FMS score^1^						
CG (*N* = 29)	11.1 (2.8)	10.5 (3.1)	−0.6 (2.4)	5.2 [4.1–6.3]	**<0.001**	0.58
IG (*N* = 37)	10.4 (2.6)	15.0 (2.2)	4.6 (2.1)			
Secondary endpoints						
TE (kg)						
CG (*N* = 29)	46.2 (17)	48.4 (18.2)	2.2 (9)	18.9 [14.1–23.9]	**<0.001**	0.46
IG (*N* = 37)	52.0 (16.6)	73.1 (20.7)	21.1 (11.3)			
TF (kg)						
CG (*N* = 29)	35.4 (15.9)	34.7 (14)	−0.7 (6.3)	12.8 [9.7–16.1]	**<0.001**	0.47
IG (*N* = 37)	38.5 (15)	50.7 (17.9)	12.2 (7.2)			
TLFl (kg)						
CG (*N* = 29)	28.6 (12.4)	30.0 (12.1)	1.4 (4.7)	13.4 [9.8–16.8]	**<0.001**	0.41
IG (*N* = 37)	34.4 (12.4)	49.2 (13.4)	14.8 (9.9)			
TLFr (kg)						
CG (*N* = 29)	29.8 (12.8)	30.6 (11.6)	0.8 (5.5)	13.2 [9.7–16.8]	**<0.001**	0.42
IG (*N* = 37)	36.4 (13)	50.4 (13.6)	14 (9.3)			
UPush (kg)						
CG (*N* = 28)	62.4 (28.4)	61.8 (29.2)	−0.6 (7.8)	17.2 [12.4–22.4]	**<0.001**	0.4
IG (*N* = 35)	73.2 (31.7)	89.8 (38.6)	16.5 (12.5)			
UPull (kg)						
CG (*N* = 28)	53.3 (22)	54.4 (21.2)	1.2 (6.4)	8.7 [5.1–12.5]	**<0.001**	0.25
IG (*N* = 35)	60.1 (23.2)	70.0 (25.8)	9.9 (8.5)			
HEl (kg)						
CG (*N* = 29)	38.1 (10.5)	43.5 (13)	5.4 (5.4)	6.7 [3.4–10]	**<0.001**	0.18
IG (*N* = 36)	41.8 (12.8)	53.8 (15.3)	12 (8.4)			
HEr (kg)						
CG (*N* = 29)	43.3 (13.8)	41.3 (13.1)	−2.1 (6)	13.4 [9.6–17.6]	**<0.001**	0.4
IG (*N* = 36)	47.2 (13.1)	58.5 (15.6)	11.3 (9.8)			
WHO‐5 score^2^						
CG (*N* = 31)	14.7 (3.3)	16.0 (4.2)	1.3 (3.6)	0.5 [−1.1 to 2.3]	0.55	0.01
IG (*N* = 39)	13.6 (4.7)	15.4 (4.3)	1.8 (3.7)			

*Note*: Mobility (FMS score), strength (kilograms), and well‐being (WHO‐5 score) values for t0, t1, and change within groups are expressed as mean (SD). Differences in change between groups are presented as mean [95% CI]. Partial η² is given for effect size.

Abbreviations: CG, control group; FMS, Functional Movement Screen; HEl, hip extension left; HEr, hip extension right; IG, intervention group; TE, trunk extension; TF, trunk flexion; TLFl, trunk lateral flexion left; TLFr, trunk lateral flexion right; UPush, upper body push; UPull, upper body pull.

^1^ A score from 0 to 21 can be achieved.

^2^ A score from 0 to 25 can be achieved.

### 3.3 Exploratory endpoints

**Figure 4:**
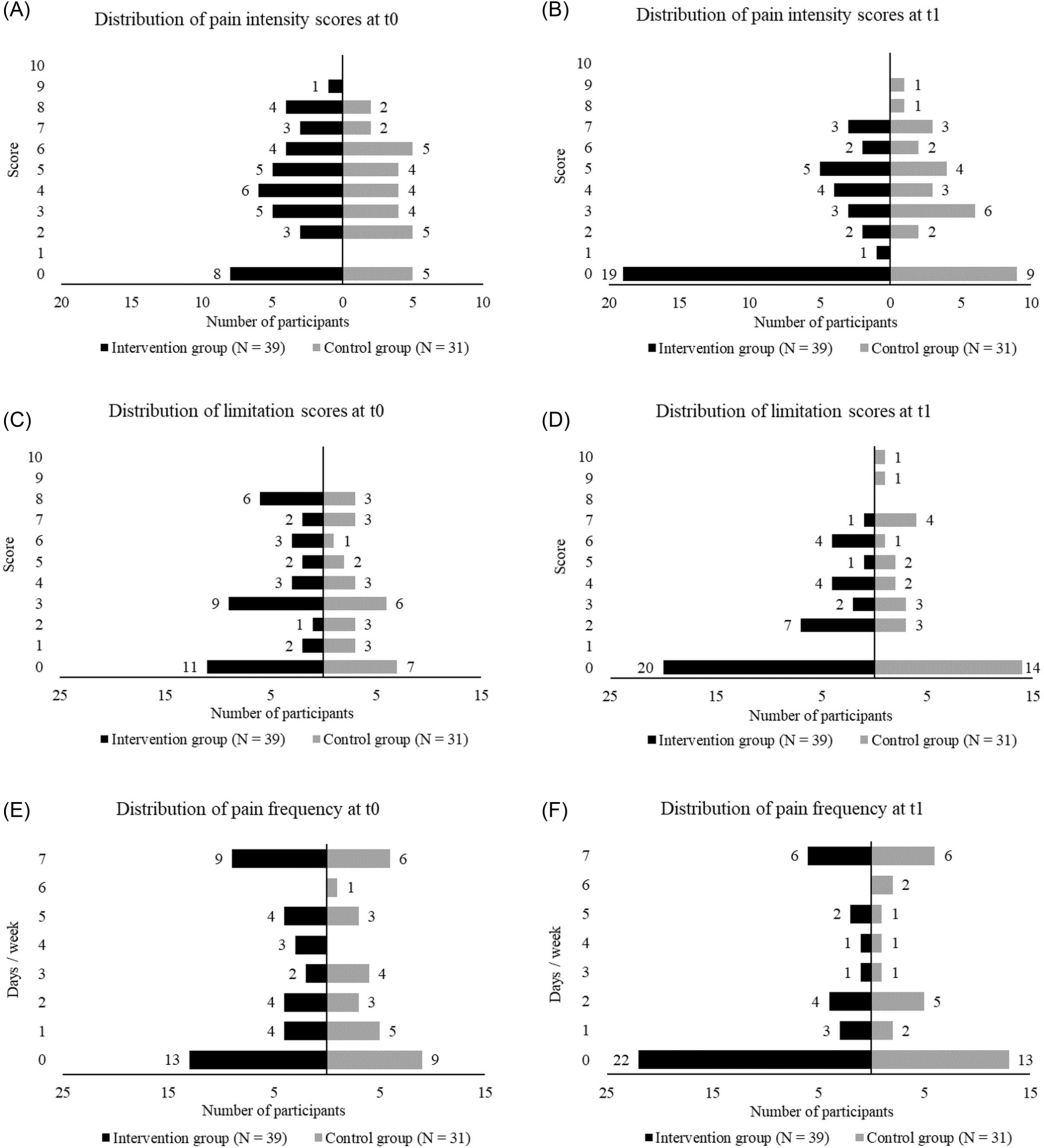
Distribution of pain intensity, limitation, and frequency for IG and CG at baseline (t0) and 6‐months follow‐up (t1). CG, control group; IG, intervention group

Regarding back issues there were no significant differences between groups at t0 for pain intensity scores (*p* = 0.75), limitation scores (*p* = 0.84), and pain frequency (*p* = 0.95). It is to mention that high proportions of both groups did not suffer from serious back issues at t0. As displayed in Figure 4, the number of pain‐ and limitation‐free individuals increased in both groups after 6 months. The decrease was higher in the IG for all measures. The mean change in pain intensity score from t0 to t1 was higher in the IG (−1.7 [2.4]) compared with CG (−0.5 [2.1]). Difference in change between groups was significant (*p* = 0.006, *r* = 0.329). Limitation scores decreased in the CG (−0.5 [2.4]) and IG (−1.6 [2.4]), resulting in a nonsignificant difference of change between groups (*p* = 0.12, *r* = 0.187). Frequency decreased in both groups with −0.3 (2.4) days/week in the CG and −1.1 (2.3) days/week in the IG. The difference of change between groups was not significant (*p* = 0.16, *r* = 0.17). Values for pain intensity, limitation, and frequency at t0 and t1 as well as change within and between groups are shown in Table 3. Medians and interquartile ranges for back‐issue data are provided in Table S5.

**Table 3:** Exploratory endpoints after 6 months for intervention (*N* = 39) and control group (*N* = 31)

Exploratory endpoints						
	t0 (baseline)	t1 (after 6 months)	Change within groups	Difference of change between groups	*p*	*r*
Pain intensity^1^						
CG	3.8 (2.4)	3.4 (2.7)	−0.5 (2.1)	−1.3 [−2.3 to −0.2]	**0.006**	0.329
IG	4.0 (2.7)	2.3 (2.5)	−1.7 (2.4)			
Limitation^1^						
CG	3.2 (2.7)	2.8 (3.1)	−0.5 (2.4)	−1.1 [−2.2 – 0]	0.12	0.187
IG	3.4 (2.9)	1.9 (2.3)	−1.6 (2.4)			
Frequency^2^						
CG	2.8 (2.7)	2.5 (2.8)	−0.3 (2.4)	−0.9 [−1.9 to0.3]	0.16	0.17
IG	2.9 (2.8)	1.8 (2.6)	−1.1 (2.3)			

*Note*: Back‐issue values at t0 and t1 as well as for change from t0 to t1 are expressed as mean (SD). Difference in change between groups is presented as mean [95% CI]. Pearson's *r* is given for effect sizes.

Abbreviations: CG, control group; CI, confidence interval; IG, intervention group; SD, standard deviation.

^1^ A score from 0 to 10 can be achieved.

^2^ Frequency is given in days per week.

## 4 DISCUSSION

The aim of the MedXFit study was to investigate the effects after 6 months of CF training on mobility, strength, and well‐being in individuals with a predominantly standing or sitting occupation that did less than two muscle and/or mobility enhancing training sessions per week prior study participation. Additionally, back issues were assessed for exploratory purposes.

After 6 months significant effects were found for mobility and strength, but not for well‐being.

In the current study, mobility was understood as proficiency to perform fundamental movement patterns in a healthy and safe manner. With the FMS, a tool was used that allows to identify deficits in flexibility as well as stability that possibly increase the risk for injuries during physical activities. At baseline FMS scores of IG (10.4 [2.6]) and CG (11.1 [2.8]) were in the range that is associated with increased injury risk (FMS score of ≤ 14).^21^, ^22^ After 6 months the CG showed slightly lower values while the IG improved, resulting in a significant difference of change in FMS scores (5.2 [4.1–6.3], *p* < 0.001). This indicates that CF improves musculoskeletal fitness and might decreases the risk for injury. Similar FMS scores (compared with t1 values of the IG) were measured among CF athletes in previous studies.^26^, ^27^ Tafuri^27^ reported an FMS score of 15.2 in experienced CF athletes (>60 months of CF training). Kaczorowska^26^ reported a score of 15.9 in CF‐athletes with at least 1 year of training experience that did not attend any specific mobility program (MobilityWOD) before.

Strength gains after CF training were confirmed by previous studies.^15^ Goins^30^ reported improvements of 12%, 13%, and 8% for repetition maxima in deadlift, back squat, and shoulder press after 6 weeks of training. Other studies observed similar results with improvements of 14.4% at front squats, 18.6% at bench presses, and 22.7% at leg presses.^31^, ^32^ Strength improvements per time in this study do not meet these values (16.5%–43% in the IG). However, comparison of this study with previous ones is difficult. Most of them were short‐term interventions with small sample sizes. Furthermore, they used complex compound movements (e.g., back squat, deadlift, and shoulder press) to measure strength.^30^, ^31^, ^33^, ^34^, ^35^ Some of these movements belong to the nine fundamental movements prescribed by CF that are trained frequently.^14^ Therefore, strength gains could partly be explained by improvements in movement proficiency in these exercises, especially when executed by inexperienced individuals during short‐term interventions. In the current study, strength was assessed under standardized conditions with isometric strength tests. These physical tasks are not directly integral in CF and thus bias caused by motor learning is minimized. Although the BC is high enough in pre‐/posttest reliability to be used in scientific research,^24^ it is hypothesized that motor learning can explain 2%–11% of improvements in the BC.^36^ Nevertheless, the difference in change between groups after 6 months in the current study supports the assumption that CF is highly effective to increase maximum isometric strength (minimum *η*² = 0.18, maximum *η*² = 0.47).

Several studies confirmed a positive association between physical activity and well‐being.^37^, ^38^ However, literature regarding the effects of CF on well‐being is still sparse and unclear.

Mean well‐being scores in this study at t0 (IG = 13.6 [4.7], CG = 14.7 [3.3]) and t1 (IG = 15.4 [4.3], CG = 16.0 [4.2]) were lower compared to previous ones. Rozada^39^ investigated 30 CF‐athletes with a mean score of 17.98. Köteles^40^ reported a mean score of 18.54. Scores might be lower because the current study included exclusively physically inactive people doing predominantly sitting or standing work. Furthermore, COVID‐19‐related restrictions might have negatively influenced well‐being.^38^ Indeed, neither Rozada^39^ nor Köteles^40^ found any positive correlations between characteristics of CF training and indicators of well‐being. In the current study both groups showed a positive change of WHO‐5 scores after 6 months, resulting in a large main effect for time (*p* < 0.001, *η*² = 0.15). A positive association between CF training and well‐being was not confirmed since the difference of change values between groups was not significant (0.5 [−1.1 to 2.3], *p* = 0.55).

CF claims to be a safe fitness program with a clear health aspect.^14^ While injury rates among CF athletes were already assessed,^19^, ^41^ long‐term intervention studies examining changes in the occurrence of back issues in CF athletes do not exist yet. The findings of the present study indicate that CF might be an appropriate training concept to reduce the occurrence. After 6 months, a greater decrease in pain intensity (−1.3 [−2.3 to −0.2]) and limitation scores (−1.1 [−2.2 to 0]), as well as pain frequency (−0.9 [−1.9 to 0.3] days/week), was observed in the IG compared with the CG. In this context, it must be mentioned that the current study was done during the COVID‐19 pandemic which could have negatively affected back‐issue measures. A multinational survey (14,975 individuals from 14 different countries) investigating mental and physical well‐being pre and during COVID‐19 restrictions observed higher musculoskeletal pain and resulting disability levels (especially prevalence of pain in the lower back, neck, and thoracic spine) when public life was restricted.^42^ It is to mention that back‐issue data were highly skewed and a significant difference between groups was found exclusively for pain intensity. Thus, results should be interpreted as exploratory.

Because of the long‐term interventional design, adherence to the training program was a major concern prior to this study. Eventually, dropout rates were lower than expected. Higher dropout in the IG (29%) compared with the CG (9%) is primarily explained by the fact, that the expense for the IG was higher.

With two CF trainings per week, participants met the recommendations for muscle and mobility enhancing activities. Additionally, cardiorespiratory training recommendations (≥75 min vigorous or ≥150 min moderate intensity) were partly accomplished. In the long run, this may inhibit the development of noncommunicable diseases.^1^, ^4^ From a physiological and individual (respectively employee) centered approach, CF can be recommended for the investigated clientele to increase mobility and strength. In fact, corporations (respectively employers) should take CF into consideration as part of their corporate health management. Potentially fewer sick days, tedious medical treatments or even disabilities may positively influence the productivity of the corporation. Embedding CF in the corporate health management of the UniBw M proved to be applicable despite highly heterogenic training groups (e.g., young soldiers, injured veterans, and civilian employees shortly before retirement) and COVID‐19‐related restrictions.

Several strengths of this study must be mentioned. First, participants were allowed to train during their working hours because the study was integrated in the corporate health management system of the UniBw M. Furthermore, gym and laboratories were on campus. Due to these factors and the given time efficiency of CF training, time expenditure was kept low which is an important factor for participation in physical activities.^43^ It is to assume that this helped to achieve the determined sample size and reduced dropout. Additionally, due to the COVID‐19 pandemic it is less likely that results were influenced by other physical activities as access to gyms and sports clubs remained rather low. Finally, training sessions were held in small groups (mostly ≤ 6 participants) by experienced CF coaches. This helped to detect and address individual, structural weaknesses, or movement flaws.

When interpreting the outcome of this study some limitations must be considered. First of all, staff and participants were not blinded. Additionally, physical activity was not measured objectively for IG and CG. Lastly, it was not assessed how participants were affected by COVID‐19 (e.g., remote work, child‐care, and hobbies) although this would have been useful for to interpret study endpoints, especially well‐being. It is recommended that future studies measure physical activity with objective methods and include assessment of lifestyle factors like alcohol consumption, stress, sleep, or diet. We estimate that future studies under normal conditions (without COVID‐19 restrictions) may see even stronger effects. To quantify the benefits of CF as part of the corporate health management, future studies should also assess sick days, productivity during working hours, and time spent for training‐related activities (transfer, hygiene, and training) during working hours.

Consistently large positive effects on mobility (*η*² = 0.58) and strength (minimum *η*² = 0.18, maximum *η*² = 0.47) in combination with the exploratory findings regarding back‐issues indicate that CF is highly beneficial to improve musculoskeletal fitness.

## 5 PERSPECTIVE

CF explicitly emphasizes the importance of physical and mental health in its fitness approach and states that most measurable values of health (blood pressure, body fat, muscle mass, etc.) can be placed on a continuum ranging from sickness to wellness to fitness – with elite athletes that are covered in advertisements, documentaries, or competition being the exception of the rule.^14^ At first instance CF should be seen as a health‐promoting training concept for anyone. Our findings support this assumption. We proved for the first time within the scope of a prospective, controlled study the broad benefits of CF for inactive individuals doing predominantly sedentary work. Participants came from a wide variety of backgrounds. Due to high scalability and versatile training stimuli, it was possible to train young soldiers alongside injured veterans or civilian employees shortly before retirement and still achieve individual adaptions. We conclude that health professionals should consider CF as a safe, efficient, and applicable training concept for individuals that are at risk for developing chronic diseases due to inactivity and sedentary behavior, especially MSD. Based on our experience, we recommend CF, particularly for heterogeneous groups. Therefore, conceivable application areas are corporate health settings and physical training of military units.

## AUTHOR CONTRIBUTIONS

**Tom Brandt**: Conceptualization; data curation; formal analysis; investigation; methodology; visualization; writing—original draft. **Annette Schmidt**: Conceptualization; methodology; writing—review & editing. **Timo Schinköthe**: Formal analysis; investigation; supervision; writing—review & editing. **Elisabeth Heinz**: Investigation. **Yannik Klaaßen**: Investigation. **Selina Limbara**: Investigation. **Marian Mörsdorf**: Investigation.

## CONFLICTS OF INTEREST

The results of this study are presented clearly, honestly, and without fabrication, falsification, or inappropriate data manipulation. The results of the present study do not constitute an endorsement by the American College of Sports Medicine. Professional relationships with companies or manufacturers who will benefit from the results of the present study do not exist. The authors declare that they have no conflict of interest.

## TRANSPARENCY STATEMENT

Tom Brandt affirms that this manuscript is an honest, accurate, and transparent account of the study being reported; that no important aspects of the study have been omitted; and that any discrepancies from the study as planned (and, if relevant, registered) have been explained.

## ETHICS STATEMENT

The study was conducted according to the guidelines of the Declaration of Helsinki and approved by the Ethics Committee of the University of the Bundeswehr Munich, Germany (06/04/2018). From all participants consent was obtained before participation in the study.

## Supporting information

Supporting information.

Supporting information.

## Data Availability

The data that support the findings of this study are available from the corresponding author upon reasonable request.

## References

[1] World Health Organization Global action plan for the prevention and control of noncommunicable diseases. World Health Organisation; 2013:103. https://www.who.int/publications/i/item/9789241506236

[2] Wessinghage T , Morsch A . Muskel‐skelett‐erkrankungen: bedeutung von bewegungsmangel und sportlicher aktivität. Pub Health Forum. 2013;21(2):21‐22. https://www.degruyter.com/document/doi/10.1016/j.phf.2013.03.020/html

[3] Finger JD , Mensink G , Lange C , Manz K . Arbeitsbezogene körperliche aktivität bei erwachsenen in deutschland. J Health Monit. 2017;2(2):29‐36. https://www.rki.de/DE/Content/Gesundheitsmonitoring/Gesundheitsberichterstattung/GBEDownloadsJ/FactSheets/JoHM_2017_02_arbeitsbezogene_koerperliche_Aktivitaet.pdf?__blob=publicationFile

[4] Garber CE , Blissmer B , Deschenes MR , et al. Quantity and quality of exercise for developing and maintaining cardiorespiratory, musculoskeletal, and neuromotor fitness in apparently healthy adults. Med Sci Sports Exer. 2011;43(7):1334‐1359. https://journals.lww.com/00005768-201107000-00026 10.1249/MSS.0b013e318213fefb 21694556

[5] Bull FC , Al‐Ansari SS , Biddle S , et al. World health organization 2020 guidelines on physical activity and sedentary behaviour. Br J Sports Med. 2020;54(24):1451‐1462. https://bjsm.bmj.com/lookup/doi/10.1136/bjsports-2020-102955 33239350 10.1136/bjsports-2020-102955 PMC7719906

[6] Finger JD , Mensink GB , Lange C , Manz K . Gesundheitsfördernde körperliche aktivität in der freizeit bei erwachsenen in deutschland. J Health Monit. 2017;2(2):37‐44. https://www.rki.de/DE/Content/Gesundheitsmonitoring/Gesundheitsberichterstattung/GBEDownloadsJ/FactSheets/JoHM_2017_02_gesundheitsfoerdernde_koerperliche_Aktivitaet.pdf?__blob=publicationFile

[7] Fuchs J , Pütz F . Prävalenz von gelenkschmerzen in deutschland. J Health Monit. 2017;2(3):66‐71. https://www.rki.de/DE/Content/Gesundheitsmonitoring/Gesundheitsberichterstattung/GBEDownloadsJ/FactSheets/JoHM_03_2017_Praevalenz_Gelenkschmerzen.pdf?__blob=publicationFile

[8] Haskell WL . Sport, exercise and health: toward the next century. Der Orthop. 2000;29(11):930‐935. http://link.springer.com/10.1007/s001320050544 10.1007/s001320050544 11149277

[9] Metter EJ , Conwit R , Tobin J , Fozard JL . Age‐associated loss of power and strength in the upper extremities in women and men. J Gerontol\ Seri A: Biol Sci Med Sci. Vol 52A, 1997:B267‐B276. https://academic.oup.com/biomedgerontology/article-lookup/doi/10.1093/gerona/52A.5.B267 10.1093/gerona/52a.5.b267 9310077

[10] Lexell J , Taylor CC , Sjöström M . What is the cause of the ageing atrophy? J Neurol Sci. 1988;84(2‐3):275‐294. https://linkinghub.elsevier.com/retrieve/pii/0022510X88901323 3379447 10.1016/0022-510x(88)90132-3

[11] RUTENFRANZ J , HETTINGER T . Studies on the dependence of physical fitness on age, sex and physical development. Zeitschr Kinderheilk. 1959;83:65‐88. http://www.ncbi.nlm.nih.gov/pubmed/14440355 14440355

[12] Vandervoort AA , Chesworth BM , Cunningham DA , Paterson DH , Rechnitzer PA , Koval JJ . Age and sex effects on mobility of the human ankle. J Gerontol. 1992;47(1):M17‐M21. https://academic.oup.com/geronj/article-lookup/doi/10.1093/geronj/47.1.M17 1730848 10.1093/geronj/47.1.m17

[13] Hollmann W , Hettinger T . Sportmedizin: Grundlagen für Arbeit, Training und Präventivmedizin. 4th ed. Schattauer; 2000:512–529.

[14] Glassman G . The CrossFit Level 1 Training Guide. CrossFit Journal. Vol 1, 3rd ed. CrossFit Incorporated; 2020:255. http://library.crossfit.com/free/pdf/CFJ_English_Level1_TrainingGuide.pdf

[15] Claudino JG , Gabbett TJ , Bourgeois F , et al. CrossFit overview: systematic review and meta‐analysis. Sports Medi ‐ Open. 2018;4(1):11. https://sportsmedicine-open.springeropen.com/articles/10.1186/s40798-018-0124-5 10.1186/s40798-018-0124-5 PMC5826907 29484512

[16] Gianzina EA , Kassotaki OA . The benefits and risks of the high‐intensity CrossFit training. Sport Sci Health. 2019 Apr 2 15(1):21‐33. Available from http://link.springer.com/10.1007/s11332-018-0521-7

[17] Eather N , Morgan PJ , Lubans DR . Improving health‐related fitness in adolescents: the CrossFit TeensTM randomised controlled trial. J Sports Sci. 2016;34(3):209‐223. http://www.tandfonline.com/doi/full/10.1080/02640414.2015.1045925 25972203 10.1080/02640414.2015.1045925

[18] Murawska‐Cialowicz E , Wojna J , Zuwala‐Jagiello J . Crossfit training changes brain‐derived neurotrophic factor and irisin levels at rest, after wingate and progressive tests, and improves aerobic capacity and body composition of young physically active men and women. J Physiol Pharmacol. 2015;66(6):811‐821. https://www.jpp.krakow.pl/journal/archive/12_15/pdf/811_12_15_article.pdf 26769830

[19] Hak PT , Hodzovic E , Hickey B . The nature and prevalence of injury during CrossFit training. J Strength Condit Res. 2013. https://journals.lww.com/00124278-900000000-97557 10.1519/JSC.0000000000000318 24276294

[20] Weisenthal BM , Beck CA , Maloney MD , DeHaven KE , Giordano BD . Injury rate and patterns among CrossFit athletes. Orthop J Sports Med. 2014;2(4):232596711453117. http://journals.sagepub.com/doi/10.1177/2325967114531177. 10.1177/2325967114531177 PMC4555591 26535325

[21] Cook G , Burton L , Hoogenboom BJ , Voight M . Functional movement screening: the use of fundamental movements as an assessment of function‐part 2. Int J Sports Phys Ther. 2014;9(4):549‐563. https://www.ncbi.nlm.nih.gov/pmc/articles/PMC4127517/ 25133083 PMC4127517

[22] Cook G , Burton L , Hoogenboom BJ , Voight M . Functional movement screening: the use of fundamental movements as an assessment of function ‐ part 1. Int J Sports Phys Ther. 2014;9(3):396‐409. https://www.ncbi.nlm.nih.gov/pmc/articles/PMC4060319/ 24944860 PMC4060319

[23] Ochs S , Froböse I , Trunz E , Lagerstrom D , Wicharz J . Einsatzmöglichkeiten und perspektiven eines neuen screeningsystems zur objektivierung des funktionszustandes der rumpfmuskulatur (IPN‐Back check). Gesundheitssport Und Spotther. 1998;14:114‐150.

[24] Schlächter K Überprüfung der Reliabilität und Validität des isometrischen Testgerätes Back Check (by Dr. Wolff) an 20‐30jährigen Probanden. [Cologne]: Deutsche Sporthochschule; 2001.

[25] Topp CW , Østergaard SD , Søndergaard S , Bech P . The WHO‐5 Well‐Being index: a systematic review of the literature. Psychother Psychosoma. 2015;84(3):167‐176. https://www.karger.com/Article/FullText/376585 10.1159/000376585 25831962

[26] Kaczorowska A , Noworyta K , Mroczek A , Lepsy E . Effect of the MobilityWOD training program on functional movement patterns related to the risk of injury in CrossFit practitioners. Acta Gymnica. 2020;50(1):3‐8. http://gymnica.upol.cz/doi/10.5507/ag.2020.002.html

[27] Tafuri S . CrossFit athletes exhibit high symmetry of fundamental movement patterns. A cross‐sectional study. muscles, ligaments and tendons. Muscles, Ligaments, Tendons J. 2016;6(1):157‐160. http://www.mltj.org/common/php/portiere.php?ID=e4af6d9f6b49189d2ffc46599fb515f7 27331045 10.11138/mltj/2016.6.1.157 PMC4915455

[28] Mitchell UH , Johnson AW , Vehrs PR , Feland JB , Hilton SC . Performance on the functional movement screen in older active adults. J Sport Health Sci. 2016;5(1):119‐125. https://linkinghub.elsevier.com/retrieve/pii/S2095254615000812 30356515 10.1016/j.jshs.2015.04.006 PMC6188618

[29] Perry FT , Koehle MS . Normative data for the functional movement screen in middle‐aged adults. J Strength Condit Res. 2013;27(2):458‐462. https://journals.lww.com/00124278-201302000-00023 10.1519/JSC.0b013e3182576fa6 22561971

[30] Goins J , Richardson MT , Wingo J , Hodges G , Leaver‐Dunn D , Leeper J Physiol Perform Effects Of Crossfit [Internet]. [Tuscaloosa]: University of Alabama. 2014. http//journals.lww.com/00005768-201405001-00835

[31] Feito Y , Hoffstetter W , Serafini P , Mangine G . Changes in body composition, bone metabolism, strength, and skill‐specific performance resulting from 16‐weeks of HIFT. PLOS One. 2018;13(6):e0198324. https://dx.plos.org/10.1371/journal.pone.0198324 29906290 10.1371/journal.pone.0198324 PMC6003684

[32] Brisebois M , Rigby B , Nichols D . Physiological and fitness adaptations after eight weeks of high‐intensity functional training in physically inactive adults. Sports. 2018;6(4):146. http://www.mdpi.com/2075-4663/6/4/146 10.3390/sports6040146 PMC6316712 30428527

[33] McKenzie MJ . Crossfit improves measures of muscular strength and power in active young females. Med Sci Sports Exer. 2015;47(5S):797. https://journals.lww.com/00005768-201505001-02465

[34] Cosgrove SJ , Crawford DA , Heinrich KM . Multiple fitness improvements found after 6‐Months of high intensity functional training. Sports. 2019;7(9):203. https://www.mdpi.com/2075-4663/7/9/203 10.3390/sports7090203 PMC6784068 31480686

[35] Crawford D , Drake N , Carper M , DeBlauw J , Heinrich K . Are changes in physical work capacity induced by high‐intensity functional training related to changes in associated physiologic measures? Sports. 2018;27 6(2):26. http://www.mdpi.com/2075-4663/6/2/26 10.3390/sports6020026 PMC6026831 29910330

[36] Dalichau S , Stein B , Schäfer K , Buhlmann J , Menken P . Effekte muskelkräftigender maßnahmen zur wirbelsäulenprotektion. B&G Bewegungsther Gesundheitssport. 2005;21(01):6‐12. http://www.thieme-connect.de/DOI/DOI?10.1055/s-2005-836295

[37] Wiese CW , Kuykendall L , Tay L . Get active? A meta‐analysis of leisure‐time physical activity and subjective well‐being. J Posit Psychol. 2018;13(1):57‐66. https://www.tandfonline.com/doi/full/10.1080/17439760.2017.1374436

[38] Wicker P , Frick B . Intensity of physical activity and subjective well‐being: an empirical analysis of the WHO recommendations. J Public Health. 2016;39(2):e19‐e26. https://academic.oup.com/jpubhealth/article-lookup/doi/10.1093/pubmed/fdw062 10.1093/pubmed/fdw062 27412174

[39] Rozada C The health aspects of CrossFitTM: Correlation analyses in everyday participants [Internet]. [Greeley]: University of Northern Colorado; 2021. Available from https://digscholarship.unco.edu/cgi/viewcontent.cgi?article=1046%26context=honors

[40] Köteles F , Kollsete M , Kollsete H . Psychological concomitants of crossfit training. Kinesiology. 2016;48(1):39‐48. https://hrcak.srce.hr/index.php?show=clanak%26id_clanak_jezik=237092

[41] Summitt RJ , Cotton RA , Kays AC , Slaven EJ . Shoulder injuries in individuals who participate in CrossFit training. Sports Health: Multidis Approach. 2016;8(6):541‐546. http://journals.sagepub.com/doi/10.1177/1941738116666073 10.1177/1941738116666073 PMC5089356 27578854

[42] Wilke J , Hollander K , Mohr L , et al. Drastic reductions in mental well‐being observed globally during the COVID‐19 pandemic. Results from the ASAP survey. Front Med. 2021;26:8. https://www.frontiersin.org/articles/10.3389/fmed.2021.578959/full 10.3389/fmed.2021.578959 PMC8032868 33842492

[43] Trost SG . Owen N, Bauman AE, Sallis JF, Brown W. correlates of adults??? Participation in physical activity: review and update. Med Sci Sports Exer. 2002;34(12):1996‐2001. http://journals.lww.com/00005768-200212000-00020 10.1097/00005768-200212000-00020 12471307

