# Corrigendum

**DOI:** 10.1002/hsr2.944

**Published:** 2022-11-18

In the article by Brandt et al.,^1^ the order of authors must be changed. The correct order is:

1. Tom Brandt

2. Elisabeth Heinz

3. Yannik Klaaßen

4. Selina Limbara

5. Marian Mörsdorf

6. Timo Schinköthe

7. Annette Schmidt

8. We apologise for the error.
