# Supplementary material for: MedXFit—Effects of 6 months CrossFit® in sedentary and inactive employees: A prospective, controlled, longitudinal, intervention study
Source: Health Sci Rep. 2022 Aug 7;5(5):e749. doi: 10.1002/hsr2.749 (PMC9358326; doi:10.1002/hsr2.749)
Supplement: Supplementary file 1 — Supporting information. [file HSR2-5-e749-s002.docx]

**Table S1** Sample training sessions: Week 1 to 26

One sample training session for every training week is displayed in the following table. Training sessions varied daily but were oriented to the week’s main emphasis. Training sessions were conducted from Monday until Friday. Training sessions were done in groups of 10 participants maximum. All participants followed the same program. Movements, intensity, and volume were scaled to individual needs by certified coaches (CrossFit Level 1 and CrossFit Level 2). Participants were instructed to train on 2 days per week.

Further explanations:

- AMRAP = As many repetitions as possible: Complete as many repetitions in the given time span.
- E(x)MOM = Every minute on the minute, x determines the length of intervals in minutes when length ≠ 1min. Complete the determined repetitions per exercise (exercises can differ for odd and even minutes) in the given time span. Rest for the remaining time of the interval.
- For time = Complete all repetitions of given exercises as fast as possible.
- Tabata = Complete 8 consecutive sets of 20sec work followed by 10sec rest.
- (x)-(x)-(x)-(x) repetitions = Complete x repetitions of every exercise, exercises are done in supersets.
- Technique = Technique training of new or complex movements.
- Odd objects = Rucksack, water container, sand sack, medicine balls, etc.

|  | Main emphasis | Warm-up | Mobility | Skill and strength | “Workout of the day” | Cool-down |
| --- | --- | --- | --- | --- | --- | --- |
| Week 1 | Lifting, carrying, and pressing of objects | 1 Round  30-40m Bear crawl  10 Ground to sky touch  30-40m Crab  10 Ground to sky touch | Integrated in warm-up | 1. Technique Deadlift, overhead press, carry with medicine ball 2. 3x10 Push up   3x30-60sec Plank | 10min AMRAP  10 Strict wall ball shot  10 Push up | Shoulder mobility/stretch |
| Week 2 | Squat | 2 Rounds  10 Hip bridge  5 Bird dog each side  5 Fire hydrant  10 Inch worm | Ankle, hip | 1. Technique Air squat, medicine ball squat, dumbbell squat, goblet squat 2. 3x10 Wall ball shot | Tabata  Wall ball shot | Hip, leg mobility/stretch |
| Week 3 | Sit up, turkish get up, step up, and box jump | TicTacToe with wall balls (2 teams, 20m between starting line and target area) | Hip, thoracic spine | 1. Technique Sit ups and turkish get up 2. Technique Step up and / or box jump | 10min AMRAP 5 Push up 10 Sit ups/heel tap 15 Air squat | Pigeon pose, cat cows, couch stretch |
| Week 4 | Rowing ergometer | 3 Rounds  Row to target (100-200m) | Hip, shoulder | 1. Technique Rowing ergometer 2. Technique Kneeling one-arm dumbbell press 3. Technique Bent over dumbbell rows 4. 1min Row for maximal distance | 10min EMOM Odd: 5 Kneeling one-arm press each side  Even: 10 Bent over dumbbell row | Back and hip mobility/stretch |
| Week 5 | Gymnastics kip | 4 Rounds  5 Slam ball deadlift  5 Slam ball clean  5 Front squat  5 Press  5 Slam ball | Thoracic spine | 1. Technique Gymnastics kip 2. Technique Wall walk | 12min AMRAP 1 Wall walk 2 Push up  3 Supermen 5 Sit-Up 7 Air-Squat | Hip, chest mobility/stretch |
| Week 6 | Barbell deadlift | 2 Rounds  30m Farmers walk  30m Suitcase walk  30m Waiters walk | Hip, ankle | 1. 30min E2MOM   5 Barbell deadlift + 5 Box jump/step up | For Time *(with partner)*  2000m Bent row  150 Squat  100 Sit up  75 Push up | Leg, chest mobility/stretch |
| Week 7 | Squat | 4-6-8 repetitions  Inch worm  Reverse snow angel  Push up  Cossack squat each side  Reverse lunge each side | Hip, ankle | 1. 30min E3MOM   5 Barbell back squat + 5 Bent row | 12min AMRAP *(with partner)*  20 Med ball clean  20 Med ball thruster  20 Sit up  20 Push up  *(start every minute with 3 Burpee)* | Leg, shoulder mobility/stretch |
| Week 8 | Rope climb | 10-8-6-4-2 repetitions  Mountain climber  Push up  Split squat  Hip bridge | Shoulder | 1. Technique rope climb 2. 5x2 Turkish get up | For Time  150 Wall ball shot  *(start every minute with 5 Sit up)* | Leg, trunk, arm mobility/stretch |
| Week 9 | Clean and press | 2 Rounds  10 Good morning  10 Walking burpee  10 Air squat  10 Jackknife | Wrist, shoulder | 1. Technique muscle clean, strict press, push press, push jerk 2. 10min EMOM   1 Clean  3 Shoulder to overhead | 10 min AMRAP  20 Slam ball  10 Box jump/step up | Shoulder, leg, hip mobility/stretch |
| Week 10 | Thruster | 2 Rounds  10 Deadlift  10 Bent row  10 Lunges  10 Goblet squat  10 Press | Ankle, hip | 1. 20min EMOM   Odd: 10 Front hold split squat  Even: 10 Body row   1. Tabata Planks   Plank with forward reach  Plank with lateral reach  Plank with forward tap  Plank with lateral tap | For Time  150 Odd object thruster  *(start every minute with 5 Sit up)* | Shoulder, trunk, leg mobility/stretch |
| Week 11 | Exercises with bodyweight and everyday objects | 4 Rounds  1 Turkish get up  5 Wind mill  10 One-arm press each side  10 One-arm bent row each side | Ankle, hip | 1. 16min AMRAP   Odd: 5 Wall ball shot  Even: 5 Box jump   1. Tabata   Superman + plank shoulder tap | 10-9-8-7-6-5-4-3-2-1repetitions  Push up  Air squat  Sit up | Shoulder, trunk, quadriceps  mobility/stretch |
| Week 12 | Split squat/pistol | 4 Rounds  5 Fire hydrant each side  5 Bird dog each side  5 Cossack squat each side  5 Inch worm | Ankle, hip | 1. Technique pistol 2. 16min EMOM   Odd: 30sec Bent row  Even: 30sec Split squat/pistol   1. Tabata   Bicycle crunch  Heel taps | 8min AMRAP 15 Odd object high pull 10 Odd object front squat 5 Odd object press/push press | Leg, arm mobility/stretch |
| Week 13 | Trunk stability | 2 Rounds  10 Good morning  10 Walking burpee  10 Air squat  10 Jackknife | Thoracic spine, shoulders | 1. Squat   3sec:3sec:3sec:0sec (down: deep-hold: up, rest)  7sec:7sec:7sec:0sec   1. 16min EMOM   Odd:30 sec Sit up  Even: 30 sec Good morning   1. Tabata   Side Plank each side | 6min AMRAP 5 Push up 10 Odd object thruster | Hip, shoulders mobility/stretch |
| Week 14 | Overhead squat | 2 Rounds  10 Good morning  10 Walking burpee  10 Air squat  10 Crunch | Ankle, hip | 1. Technique Overhead squat 2. Tabata   Overhead squat hold in bottom position   1. 8min EMOM   2 Turkish get up | 5min AMRAP  21 Overhead squat  15 Odd object bent row  9 Burpee  --- Rest 2 min----  5min AMRAP  9 Overhead squat  15 Odd object bent row  21 Burpee | Lg, thoracic spine, shoulders mobility/stretch |
| Week 15 | Ground to overhead | 2 Rounds  10 Good morning  10 Walking burpee  10 Air squat  10 Crunch | Ankle, hip | 1. Technique high pulls 2. Technique ground to overhead variations 3. Tabata   Sit up   1. Tabata   L-sit hold   1. Tabata   Russian twist | 50-40-30-20-10  Odd object ground to overhead  Odd object bent row | Back, hips mobility/stretch |
| Week 16 | Air Squat | 2 Rounds  10 Odd object deadlift  10 Odd object bent row  10 Odd object lunge  10 Odd object goblet squat  10 Odd object press | Walking world greatest stretch | 1. 16 Min EMOM   Odd: 30sec Wall climb  Even: 30sec Body row   1. 10 Push up 3sec:3sec:3sec:0sec (down: deep-hold: up: rest)   10 Deadlift 3sec:3sec:3sec:0sec  3 Push up 6sec:6sec:6sec:0sec  3 Deadlift 6sec:6sec:6sec:0sec | For Time  200 Air squat  *(Start every minute starts with 3 Burpee)* | Leg, hip, shoulder mobility/stretch |
| Week 17 | Box jump over | 3 Rounds  10 Squat into knee hug  10 Walking burpee  10 Crunch | Hip | 1. Technique box jump over 2. 12min EMOM (30sec work each minute)   Odd: Dumbbell overhead squat  Even: Superman   1. Tabata   Side plank left / right | 5 Rounds  10 Burpee  10 Sit up  10 Reverse lunge  10 Push up | Hip, shoulder  mobility/stretch |
| Week 18 | Squat | 2 Rounds (PVC pipe)  10 Snatch grip deadlift  10 Snatch grip high pull  10 Snatch grip press behind the neck  10 Overhead lunges  10 Squats | Shoulder, hip, ankle | 1. Technique Squat variations 2. 10 Slow deadlift 3sec:3sec:3sec:0sec (down:deep-hold:up:rest)   10 Slow row 3sec:3sec:3sec:0sec  3 Slow Deadlift 10sec:10sec:10sec:0sec  3 Slow Row 10sec:10sec:10sec:0sec | EMOM for AMRAP  1. Slam ball thruster  2. Slam ball over shoulder  3.Slam ball  -2 minutes rest-  1. Slam ball thruster  2. Slam ball over shoulder  3. Slam ball | Hip, legs, thoracic spine  mobility/stretch |
| Week 19 | Jump rope | 3 Rounds  20 Lunge  15 Tuck up  10 Inch worm | Hip, ankle | 1. Technique Jump rope 2. Tabata   Overhead squat hold  Superman   1. Tabata   Hip Bridge  Hollow Hold | 12min AMRAP  20 Jumping lunge (scale: lunge)  15 Commando plank  10 Odd object deadlift  5 Sit up | Hip, trunk mobility/stretch |
| Week 20 | Barbell deadlift | 2 Rounds (PVC pipe)  10 Snatch grip deadlift  10 Snatch grip high pull  10 Snatch grip press behind the neck  10 Overhead lunge  10 Squats | Hip | 1. Technique barbell deadlift 2. 12min E2MOM   8 Deadlift (increase weight each round)   1. Tabata Hollow Hold | 21-18-15-12-9 repetitions  Box Jump over  Lunges | Hip, legs mobility/stretch |
| Week 21 | Pull up | 2 Rounds  10 Sit up  10 Push up  10 Squat  10 Shoulder to overhead  10 Overhead squat | Shoulder, thoracic spine | 1. Technique pull up variations 2. Tabata   Hollow hold  Superman   1. 10min EMOM   Odd: 10 Wall angel  Even: 10 Push up | Chipper  50 Sit up  40 (jumping) Lunge  30 Wall ball shot  20 Mountain climber  10 Box jump/step up (walking)  burpee | Back, shoulder, chest mobility/stretch |
| Week 22 | Rope climb | 2 Rounds 5 Walk out + Push up 10 Plank shoulder tap  15 Goblet squat  10 Walking burpee 5 Swimmer | Shoulder, hip | 1. 20min EMOM 1. 1- 3 Rope climb   2. 10-15 Push up + alternating toe touch 3. 30sec Side plank pulses left 4. 30sec Side plank pulses right   1. Tabata   Sit Ups Heel taps  Slam ball bent rows | 10min AMRAP  9 Slam ball thruster  15 Burpee over slam ball  21 Wall ball shot | Leg, hip, shoulder mobility/stretch |
| Week 23 | Thruster,  Pull up | 2 Rounds:  10 Walking burpee  10 Odd object Row  10 Squat  10 Odd object press | Shoulder, hip | 1. 16min EMOM   Odd: 30sec Odd object thruster  Even: 30sec Odd object bent row   1. Tabata   Crunch  Wall sit | Chipper  10 Push up  20 Mountain climber  30 Sit up  40 Lunge  50 Jumping jack | Shoulder, hip  mobility/stretch |
| Week 24 | Muscle clean and push press | 2 Rounds with a PVC-Pipe 10 Snatch grip deadlift  10 Snatch grip high pull  10 Snatch grip press behind the neck  10 Overhead lunge  10 Overhead squat | Shoulder, hip | 1. Technique Muscle clean 2. Technique Push press 3. Tabata   Side plank | For time  50 Burpee  *(start every minute starts with 25 Jump rope single under)* | Shoulder, hip, leg mobility/stretch |
| Week 25 | Barbell deadlift | 2 Rounds  10 Good morning  10 Squats into side bend  20 Arm circle  10 Barbell bent row | Shoulder, hip | 1. Technique Barbell deadlift 2. E3MOM24   6-10 Deadlift + 20 Dead bug | 12min AMRAP  5 Push up  10 Squat  15 Seated leg raise over object  20 Jump rope single under | Hip, shoulder  mobility/stretch |
| Week 26 | Sumo deadlift high pull | 3 Rounds  10 Good morning  20 Arm circle  10 Odd object bent row | Shoulder, hip | 1. Technique Sumo deadlift high pull 2. Tabata   Superman pulse   1. Tabata   Side plank | 1. 2 Rounds a) 1 Minute Wall sit b) EMOM 4  12 Lunge 2. For time 150 Odd Shoulder to overhead   *(start every minute with 2 Push up + 8 Shoulder tap)* | Hip, leg, shoulder mobility/stretch |
