# Supplementary material for: MedXFit—Effects of 6 months CrossFit® in sedentary and inactive employees: A prospective, controlled, longitudinal, intervention study
Source: Health Sci Rep. 2022 Aug 7;5(5):e749. doi: 10.1002/hsr2.749 (PMC9358326; doi:10.1002/hsr2.749)
Supplement: Supplementary file 2 — Supporting information. [file HSR2-5-e749-s001.docx]

**Table S2** Normal distribution of mobility (FMS score), strength (Dr. WOLFF BackCheck® 617), well-being (WHO-5 score), and back issues (pain intensity, limitation and pain frequency) at baseline (t0), after 6 months (t1) and for change (t1 - t0) analyzed with Kolmogorov-Smirnov and Shapiro-Wilk test; p is given for all tests.

Abbreviations: IG = intervention group, CG = control group, TE = trunk extension, TF = trunk flexion, TLFl = trunk lateral flexion left, TLFr = trunk lateral flexion right, UPush = upper body push, UPull = upper body pull, HEl = hip extension left, HEr = hip extension right

|  | Group | Kolmogorov-Smirnov | | | Shapiro-Wilk | | |
| --- | --- | --- | --- | --- | --- | --- | --- |
|  |  | t0 (baseline) | t1 (after 6 months) | Change (t1 - t0) | t0 (baseline) | t1 (after 6 months) | Change (t1 - t0) |
| FMS score | CG | .2 | .154 | .006 | .432 | .470 | .021 |
|  | IG | .141 | .038 | .007 | .717 | .231 | .046 |
| TE | CG | .054 | .151 | .2 | .02 | .045 | .643 |
|  | IG | .038 | .089 | .134 | .042 | .087 | .037 |
| TF | CG | .2 | .085 | .011 | .265 | .295 | < .001 |
|  | IG | .2 | .092 | .196 | .118 | .023 | .069 |
| TLFl | CG | .115 | .038 | .2 | .064 | .004 | .204 |
|  | IG | .2 | .013 | .2 | .374 | .038 | .639 |
| TLFr | CG | .2 | .068 | .2 | .203 | .006 | .508 |
|  | IG | .2 | .2 | .2 | .176 | .251 | .453 |
| UPush | CG | .134 | .2 | .2 | .064 | .138 | .670 |
|  | IG | .013 | .114 | .2 | .011 | .037 | .767 |
| UPull | CG | .2 | .2 | .097 | .374 | .305 | .390 |
|  | IG | .2 | .122 | .066 | .159 | .096 | < .001 |
| HEl | CG | .2 | .2 | .2 | .409 | .250 | .297 |
|  | IG | .01 | .167 | .140 | .001 | .048 | .181 |
| HEr | CG | .2 | .188 | .2 | .034 | .002 | .718 |
|  | IG | .2 | 0.2 | .2 | .391 | .588 | .216 |
| WHO-5 score | CG | .199 | .002 | .138 | .617 | .008 | .650 |
|  | IG | .097 | .2 | .2 | .009 | .169 | .612 |
| Pain intensity | CG | .2 | .012 | .022 | .101 | .016 | .112 |
|  | IG | .075 | < .001 | .013 | .023 | < .001 | .068 |
| Limitation | CG | .09 | < .001 | < .001 | .008 | < .001 | .009 |
|  | IG | .012 | < .001 | .018 | < .001 | < .001 | .034 |
| Pain frequency | CG | .004 | < .001 | < .001 | < .001 | < .001 | .006 |
|  | IG | .002 | < .001 | < .001 | < .001 | < .001 | .006 |

**Table S3** P-values for differences between groups at baseline (t0) and after 6 months (t1) for mobility (FMS score), strength (Dr. WOLFF BackCheck® 617), and well-being (WHO-5 score) as well as p-values and effect sizes (Cohen´s d) for differences in change from t0 to t1 between groups analyzed with independent t-test (BCa; samples N = 1000).

Abbreviations: TE = trunk extension, TF = trunk flexion, TLFl = trunk lateral flexion left, TLFr = trunk lateral flexion right, UPush = upper body push, UPull = upper body pull, HEl = hip extension left, HEr = hip extension right

|  | t0 | t1 | Change (t1 - t0) | |
| --- | --- | --- | --- | --- |
|  | p | p | p | d |
| FMS score | .349 | <.001 | <.001 | 2.32 |
| TE | .173 | <.001 | <.001 | 1.82 |
| TF | .423 | <.001 | <.001 | 1.88 |
| TLFl | .06 | <.001 | <.001 | 1.67 |
| TLFr | .047 | <.001 | <.001 | 1.68 |
| UPush | .146 | .004 | <.001 | 1.61 |
| UPull | .238 | .011 | <.001 | 1.13 |
| HEl | .216 | .005 | <.001 | .93 |
| HEr | .251 | <.001 | <.001 | 1.61 |
| WHO-5 score | .239 | .567 | .547 | .15 |

**Table S4** P-values for differences between groups at baseline (t0) and after 6 months (t1) for mobility (FMS score), strength (Dr. WOLFF BackCheck® 617), and well-being (WHO-5 score) as well as p-values and effect sizes (Pearson's r) for differences in change from t0 to t1 between groups analyzed with Mann-Whitney-U-Test.

Abbreviations: TE = trunk extension, TF = trunk flexion, TLFl = trunk lateral flexion left, TLFr = trunk lateral flexion right, UPush = upper body push, UPull = upper body pull, HEl = hip extension left, HEr = hip extension right

|  | t0 | t1 | Change (t1 - t0) | |
| --- | --- | --- | --- | --- |
|  | p | p | p | r |
| FMS score | .346 | <.001 | <.001 | .82 |
| TE | .127 | <.001 | <.001 | .73 |
| TF | .356 | <.001 | <.001 | .75 |
| TLFl | .047 | <.001 | <.001 | .67 |
| TLFr | .044 | <.001 | <.001 | .68 |
| UPush | .180 | .004 | <.001 | .66 |
| UPull | .323 | 0.34 | <.001 | .52 |
| HEl | .322 | .006 | <.001 | .47 |
| HEr | .105 | <.001 | <.001 | .68 |
| WHO-5 score | .553 | .473 | .573 | .07 |

**Table S5** Median and interquartile ranges (IQR) of back-issue values at baseline (t0) and after 6 months (t1) as well as median and IQR of change within groups (t1 - t0).

|  | t0  (baseline) | t1  (after 6 months) | Change within groups  (t1 - t0) |
| --- | --- | --- | --- |
| Pain intensity^1^  CG  IG | 4 (4)  4 (4) | 3 (5)  1 (5) | 0 (2)  -2 (3) |
| Limitation^1^  CG  IG | 3 (4)  3 (6) | 2 (5)  0 (4) | -1 (2)  -1 (3) |
| Frequency^2^  CG  IG | 2 (5)  2 (5) | 2 (6)  0 (3) | 0 (2)  0 (2) |

Abbreviations: CG = control group, IG = intervention group.

^1^ A score from 0 to 10 can be achieved.

^2^ Frequency is given in days per week.
